# Night flight facilitates late breeding catch-up in a long-distance migratory seabird

**DOI:** 10.1038/s41598-024-82328-4

**Published:** 2024-12-30

**Authors:** Katrina Siddiqi-Davies, Joe Wynn, Oliver Padget, Sarah Bond, Jóhannis Danielsen, Annette L. Fayet, Lewis Fisher-Reeves, Robin Freeman, Natasha Gillies, Holly Kirk, Lou Maurice, Greg Morgan, Martyna Syposz, Akiko Shoji, Tim Guilford

**Affiliations:** 1 Department of Biology, University of Oxford, Mansfield Road, Oxford, OX1 3SZ UK; 2 Institut Für Vogelforschung “Vogelwarte Helgoland”, An Der Vogelwarte 21, 26386 Wilhelmshaven, Germany; 3 Department of Earth, Ocean and Ecological Sciences, University of Liverpool, Jane Herdman Building, Liverpool, L69 3GP UK; 4 Faroe Marine Research Institute Nóatún 1, P.O. Box 305, FO 110 Tórshavn, Faroe Islands; 5 Norwegian Institute for Nature Research, Høgskoleringen, 7034 Trondheim, Norway; 6 Zoological Society of London, London, NW1 4RY UK; 7 ICON Science, Royal Melbourne Institute of Technology, La Trobe Street, Melbourne, VIC 3000 Australia; 8 British Geological Survey, MacLean Building, Benson Ln, Crowmarsh Gifford, Wallingford, OX10 8ED UK; 9 Royal Society for the Protection of Birds, Pembrokeshire Coast National Park, Haverfordwest, SA62 6PY UK; 10 Department of Vertebrate Ecology and Zoology, Faculty of Biology, University of Gdańsk, Wita Stwosza 59, 80-308 Gdańsk, Poland; 11 Graduate School of Environmental Studies, Nagoya University, Nagoya, 464-8601 Japan

**Keywords:** Migration, Phenology, Optimisation, Behaviour, Biotelemetry, Seabird, Animal migration, Behavioural ecology, Animal behaviour

## Abstract

Long-distance migrants must optimise their timing of breeding to capitalise on resources at both breeding and over-wintering sites. In species with protracted breeding seasons, departing earlier on migration might be advantageous, but is constrained by the ongoing breeding attempt. Here we investigated how breeding timing affects migratory strategies in the Manx shearwater (*Puffinus puffinus*), a trans-hemispheric migratory seabird with large temporal variation in the onset of breeding. Using a geolocator tracking dataset, we found that that later-laying shearwaters had shorter overall breeding periods, yet still departed later for autumn migration. Earlier laying birds had increased migratory duration, stopped with greater frequency and at sites of higher chlorophyll concentration. Meanwhile, later departing birds flew more at night during migratory stints, and night flight generally increased with moon illumination, which could reflect moonlight providing the light conditions required for visually guided flight. Accordingly, birds that experienced higher levels of moon illumination whilst migrating had shorter migration durations. Here we provide an example of migratory behaviour being adjustable with breeding timing, allowing birds to both complete breeding and capitalise on resource availability at the wintering site.

**Supplementary Information:**

The online version contains supplementary material available at 10.1038/s41598-024-82328-4.

## Introduction

Post-breeding migration in high latitude animals is considered to be an adaptation to shifting but predictable seasonal resources^1,2^. When freed from the constraints of breeding, birds migrate to regions of higher seasonal productivity, sometimes travelling thousands of kilometres^3–5^. Timing migration to the wintering grounds requires optimising several trade-offs. For example, life history trade-offs may exist such as whether the bird stays to finish the breeding attempt and have the best chance of fledging healthy offspring, or instead leaves early to maximise winter resource acquisition^2,6^. Equivalently, trade-offs occur during migration and may be based on the spatiotemporal distribution of resources such as the timing of the decline of breeding site resources and the expected increase in non-breeding site resource availability, the speed of migratory flight, the potential for refuelling along the way or, conversely, travel over unsuitable habitat or in adverse weather conditions^7–9^. Natural selection positions animals at the points along trade-off axes that on average maximise the net benefit^10^. While migrating early might position a bird better with respect to resources, this may not be a fitness-maximizing strategy if it is at the cost of abandoning the current breeding attempt (presumably chick death and failure ensue). Hence, later-breeding birds may need to find other ways of mitigating for late departure. However, other mechanisms that have evolved to help birds ‘catch up’ and reach areas of higher seasonal resource abundance are poorly understood^11,12^.

The study of such optimisation in migration, first suggested by Alerstam (1991), has led to research in migration strategy incorporating an optimisation perspective^13,14^. Assisted by technological advances in biotelemetry, we have gained insight into some of the ways in which long-distance migrants optimise migration, timing departure with favourable environmental conditions and stopping at productive foraging sites^15–17^. For example, we have seen evidence in multiple long distance songbird species, that late-nesting individuals, despite delays in migratory departure, do not arrive later at their over-wintering grounds^18–20^. However, despite taking some of the furthest documented avian migrations, and having some of the most constraining and protracted breeding periods, studies into optimisation of migration phenology in seabirds are scarce^21,22^. Whilst some birds complete their migration in a single bout of flight, the majority break up their journey with a series of stopovers^3^. Stopovers can have multiple functions but are considered to be primarily for refuelling^8^. A study of Cory’s shearwaters *Calonectris borealis* found that birds departing earlier on migration took longer to reach the over-wintering grounds and had more stopovers than later birds^22^. This suggests that phenology might cause differences in migration behaviour amongst individuals, with earlier birds being able to take advantage of foraging opportunities *en route*, whilst later birds prioritise migration speed. When migrating between stopovers, the Cory’s shearwaters were shown to fly both by day and night, flying more on nights of higher moon illumination^22^. In shearwaters, which are nocturnal when on land, moonlight has been shown to reduce breeding season colony attendance, with birds favouring darker, less risky nights to avoid predation^23,24^. However, predation risk to seabirds at sea is presumably low and moonlight may instead provide an opportunity to make use of night flight during migration stints^25^. Migrants that use visual cues for flight appear constrained in their ability to migrate at night by darkness, and therefore may utilise nights with greater moon illuminance to catch up on migration^26,27^.

Manx shearwaters *Puffinus puffinus* are procellariform seabirds that take a long-distance, trans-equatorial migration, breeding at colonies based predominantly around the British Isles and Ireland, and over-wintering around the Patagonian shelf^28^. This migration has been characterised in some detail, with north-bound migrations known to take 14–44 days, with birds stopping for an average of 8 days, mainly at sites clustered off southeast Brazil in areas of high net primary productivity^28,29^. Despite evidence that earlier laying birds rear fledglings with greater survival rates, there is approximately one month of variation in lay dates in Manx shearwaters, an effect unattributable to clutch size as shearwaters lay a single egg per breeding season.^30^ It follows therefore that we might expect similar variation in the time at which adult birds will finish chick provisioning and depart for migration^31^. Accordingly, previous work has documented at least 20 days of variation in migratory departure dates^29^. As long-distance migrants with individual variation in breeding schedules and a high number of stopovers when compared to other seabirds^29,32^, Manx shearwaters are therefore a suitable species in which to investigate optimisation of migratory phenology. Here, we aim to establish whether variation in migration start date is caused by differences in lay date between individual birds and—given the behaviour of the closely-related Cory’s shearwater^22^—whether early and late departing birds vary their migratory strategies to compensate for variation in breeding phenology. This we assessed using a large 14-year dataset of geolocator-derived Manx shearwater migratory movements, with which we aim to provide a detailed exploration of how migratory behaviour may vary within a species.

## Results

### Migration metrics

**Fig. 1:**
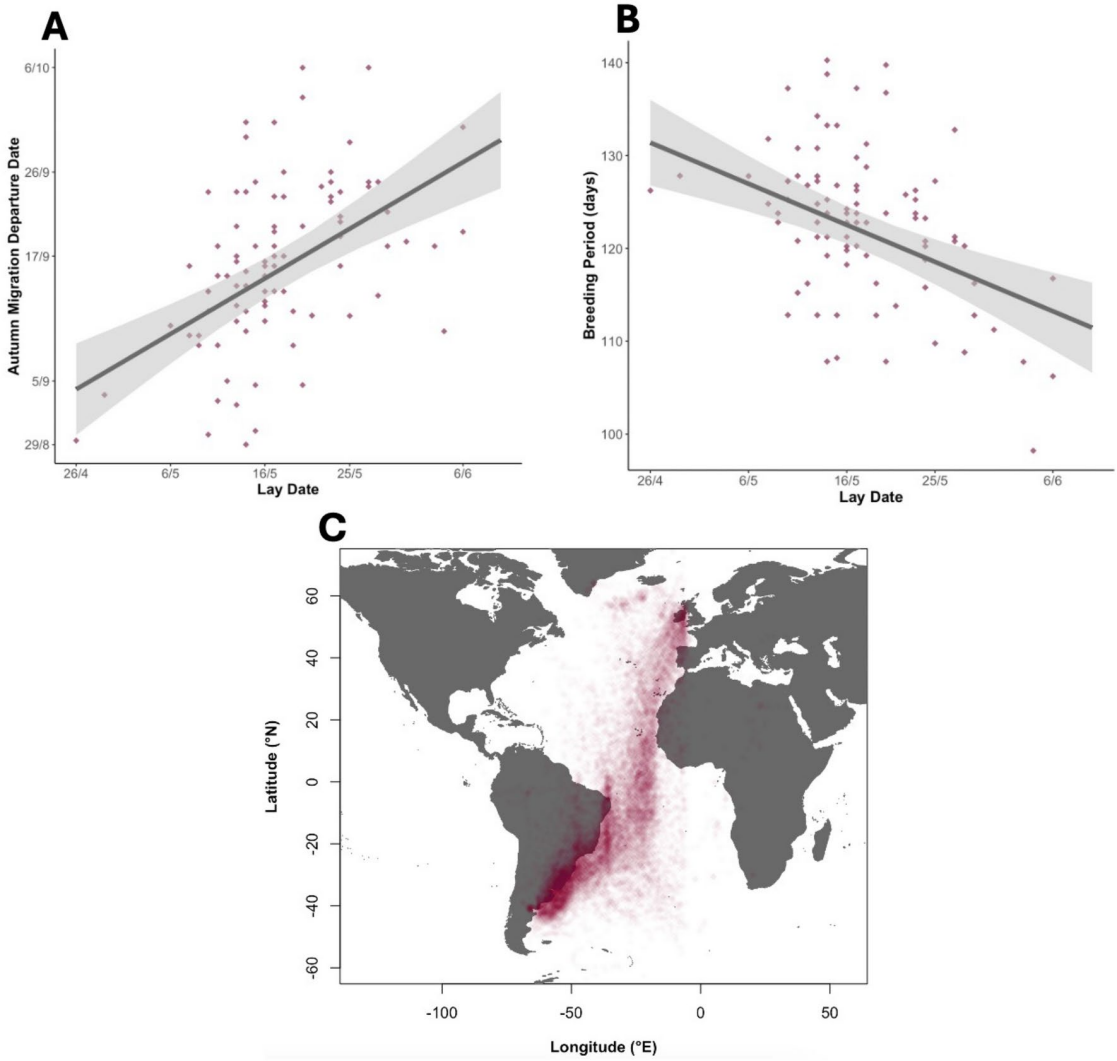
Visualising autumn migration and its relationship with breeding phenology. (**A**) Lay date plotted against GLS-determined autumn migration date (n = 89; Skomer birds only). The regression line is derived from our linear mixed-effects model (see Table 2). (**B**) Lay date plotted against breeding period (the number of days between lay date and GLS determined autumn migration date (n = 89; Skomer birds only). (**C**) The post-breeding migration of 351 shearwaters plotted with transparency from GLS derived position estimates.

All birds migrated southbound to the Patagonian shelf (Fig. 1C). We identified 1,402 stopovers of varying lengths in 351 tracks, with stopovers being present in all tracks (Table 1). The mean continuous stopover length was 2.69 ± SD 1.53 days, with the longest stopover lasting 22 days. On average birds stopped for 10.07 ± SD 6.3 days over a migration. The mean migration start date was September 14^th^ and the mean migration end date the 16th of October. The mean migrating time between stopovers was 5.63 ± 3.04 days and the mean number of continuous hours of flight was 0.88 ± 0.08 for all birds. However, birds could fly much longer, with the longest period of non-stop continuous flight recorded being 21 h. Remarkably, one bird migrated from Wales to South America in just 12 days (Table 1).

**Table 1:** Summary statistics of autumn migration parameters.

	Mean	Minimum	Maximum
Migration start date	September 14th	August 8th	October 11th
Migration end date	October 16th	September 11th	December 4th
Migration duration	31.72 ± 8.49 days	12 days	76 days
Stopover number	4.04 ± 2.1	1	12
Total stopover days	10.07 ± 6.30 days	1	44
Stopover length	2.69 ± 1.53 days	1 day	22 days
Time between stopovers	5.63 ± 3.04 days	1 day	23 days
Continuous flight	0.88 ± 0.08 h	0.16 h	21
Lay date (n = 89)	May 17th	26th April	6th June

For all Parameters we present the mean (of individual means) ± the standard deviation, and minimum and maximum values derived from 351 bird migrations (except in the case of lay date where n = 89).

### Effects of phenology and moonlight on behaviour

**Fig. 2:**
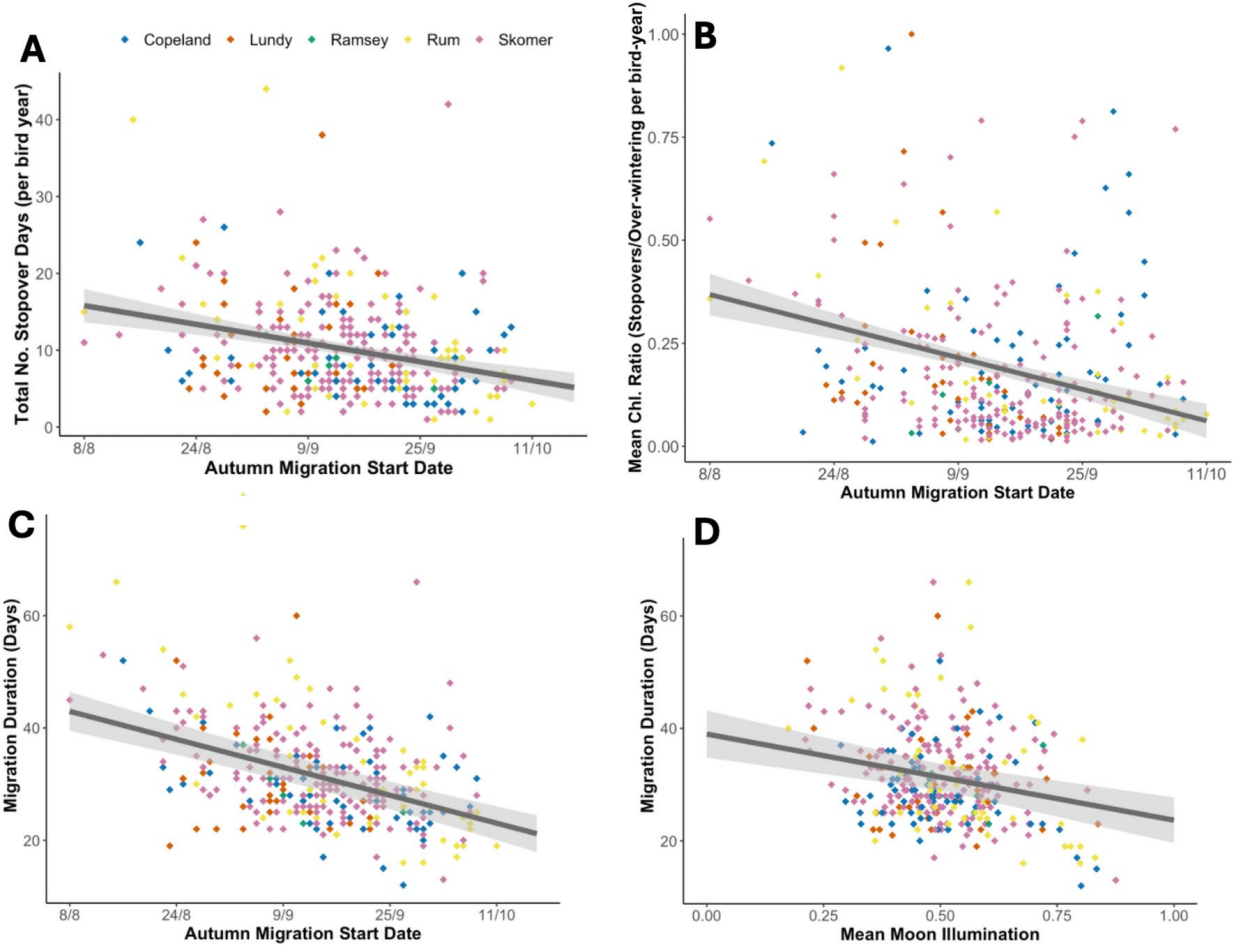
The effect of autumn migration date and moon illumination on migratory phenology and stopover productivity. (**A**) The total number of stopover days plotted against autumn migration departure date (n = 351). (**B**) Mean ratio of chlorophyll at stopover sites/the overwintering site (the Patagonian shelf) against migratory start date (n = 351). (**C**) Migration duration plotted against the migratory start date (n = 351). (**D**) Migratory duration plotted against mean moon illumination (n = 351). In all plots the regression line is derived from the linear mixed-effects model (see Table 2), and each point represents a single bird year, with points coloured to represent the 5 different colonies.

We used Likelihood Ratio Tests and bootstrapped confidence intervals to evaluate the interactive, independent and random effects of models. For Skomer birds – where lay date data were available—a 1-day increase in lay date increased migration departure significantly by 0.6 days (β = 0.57, 95%CI [0.37, 0.75], χ^2^c = 27.35, *p* < 0.0001; Fig. 1; model 8). However, the total breeding period decreased by 0.4 days for every one day increase in lay date, implying that later-laying birds spend less time providing parental care for their chick (β = − 0.44, 95%CI [− 0.64, − 0.25], χ^2^c = 18,09, *p* < 0.0001; Fig. 1; model 9). A later start date of autumn migration also significantly reduced the total number of stopover days observed on migration, with the number of stopover days decreasing by 0.16 for every 1-day delay in autumn migration start date (β = − 0.16, 95%CI [− 0.218, − 0.097], χ^2^c = 28.53, *p* < 0.0001; Fig. 2; model 5). As autumn migratory start date got later, the daily number of hours spent foraging at each stopover location did not vary (β = 0.01, 95%CI [− 0.007, − 0.030], χ^2^c = 0.3, *p* < 0.05; model 6). However, the mean chlorophyll ratio between stopover and over-wintering sites showed significant variation with autumn migratory start date (β = − 0.01, 95%CI [− 0.007, − 0.030], χ^2^c = 42.316, *p* < 0.0001; Fig. 2; model 10). Therefore, later departing birds stopped at sites with a lower concentration of chlorophyll relative to the over-wintering grounds.

**Fig. 3:**
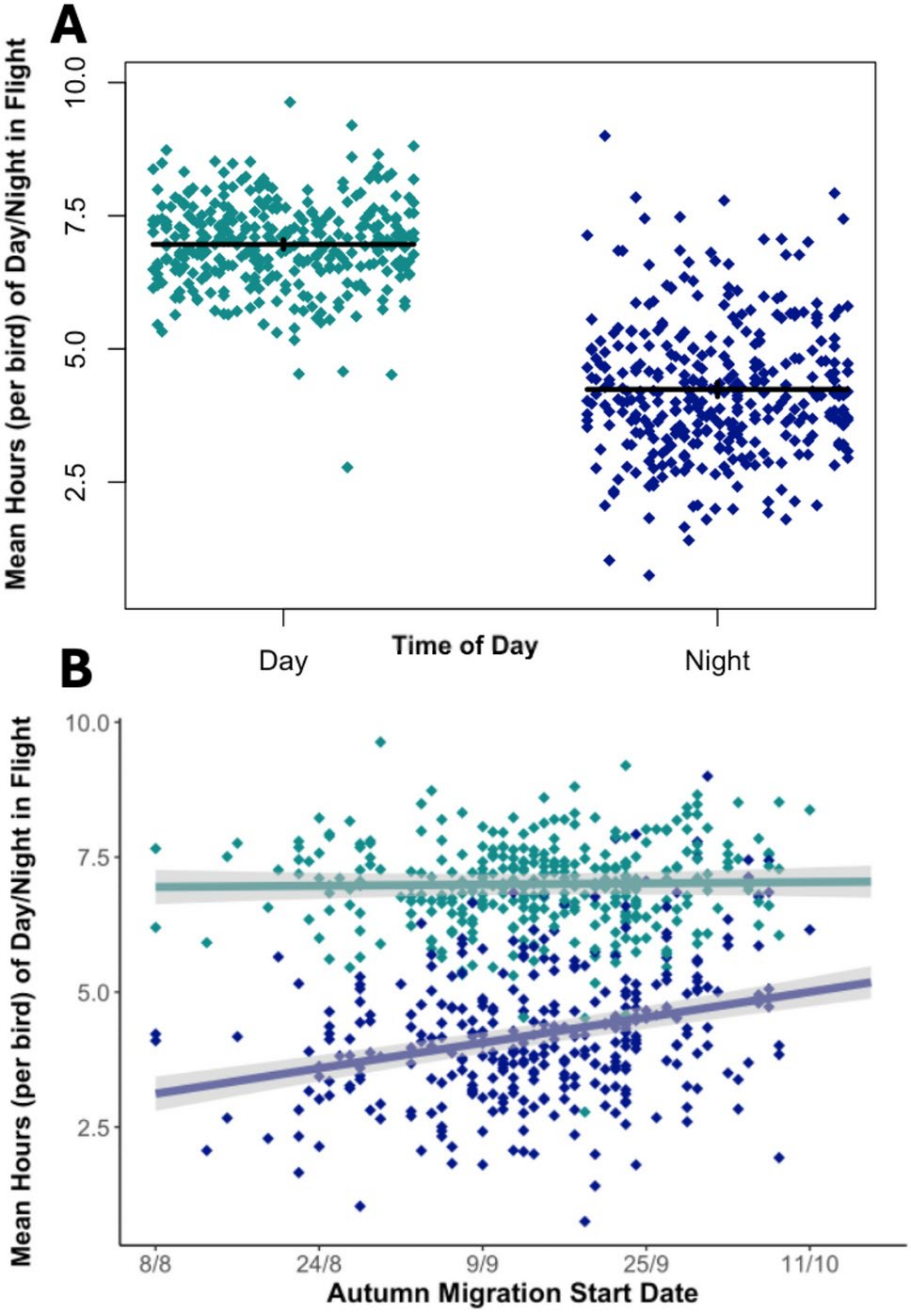
The number of hours spent in day and night flight during migration. (**A**) Mean number of hours spent in flight per day and night plotted with horizontal ‘jitter’. Solid black lines represent the population mean ± the 95% confidence interval. (**B**) Mean number of hours spent in flight for day (teal) and night (purple) plotted against autumn migration start date. The regression line and confidence intervals are derived from the linear mixed-effects model (see Table 2). In both plots, each point represents the mean of a single bird year.

**Fig. 4:**
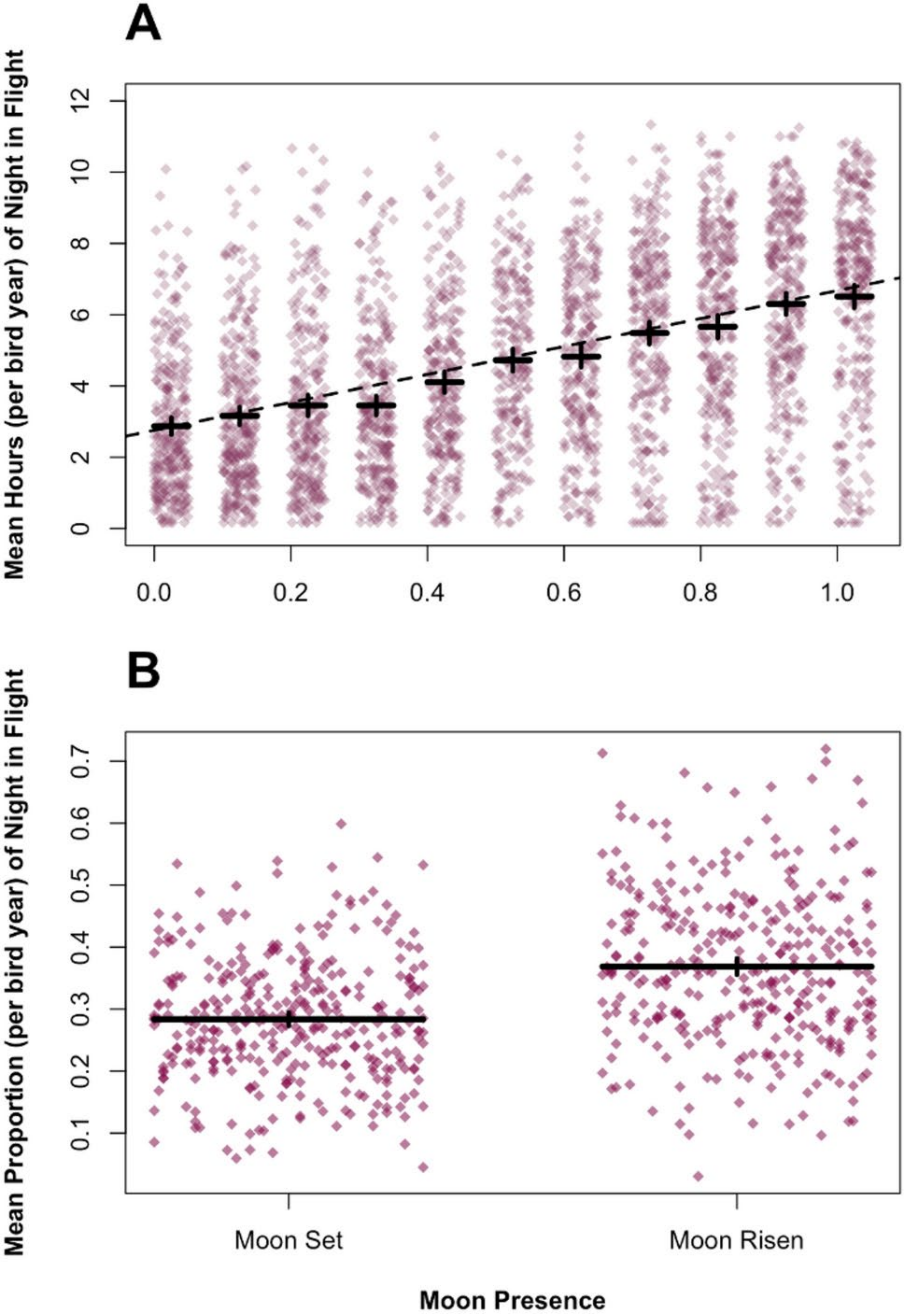
The effect of moonlight on the numbers of hours spent flying at night (n = 351). (**A**) The mean number of hours at night spent in flight by proportion of moon illumination (0–1). The regression line is derived from the linear mixed-effects model (see Table 2). (**B**) The effect of moon presence on the mean proportion of hours of night flight. Both plots are plotted with horizontal ‘jitter’ and black solid lines represent the overall mean for all birds ± the 95% confidence interval whilst each point represents the mean of a single bird year.

Birds flew for significantly fewer hours during the night than the day (β = − 3.82, 95%CI [− 4.14, − 3.50], χ^2^c = 2656.1, *p* < 0.0001; Fig. 3). There was, additionally, a significant interaction between day/night and migration start date on the hours of flight per day/night (β = 0.03, 95%CI [0.02, 0.04], χ^2^c = 42.53, *p* < 0.0001; Fig. 3; model 1), meaning that birds leaving later on migration flew more at night. Both the proportion of moonlight (from 0–1 with 1 being a full moon) (β = 3.25, 95%CI [2.58, 3.90], χ^2^c = 1247.1, *p* < 0.0001; Fig. 4; model 2) and later migration start (β = 0.015, 95%CI [0, 0.03], χ^2^c = 20.10, *p* < 0.0001; Fig. 4; model 2) significantly increased the number of hours of night flight during nights when birds were migrating. However, the interactive effect of moonlight and migration start date was not statistically significant (β = 0.02, 95%CI [0.01, 0.04], χ^2^c = 1.85, *p* > 0.5; Fig. 4; model 2), suggesting that the effect of moonlight did not differ between early and late-migrating birds. Birds flew for a higher proportion of the night when the moon was above the horizon (β = 0.08, 95%CI [0.07, 0.1, χ^2^c = 140.52, *p* < 0.0001; Fig. 4; model 3). The number of hours spent in behaviour we classified as foraging at night during migration was small, and decreased with moonlight (β = − 2.85, 95%CI [− 3.37, − 2.30, χ^2^c = 1069.3; *p* < 0.0001; model 4), whilst no statistical effects were found for migration start date (β = 0, 95%CI [− 0.01, 0.01, χ^2^c = 0.82, *p* > 0.05; model 4), or the interactive effect of moonlight and migration start date on night time foraging (β = − 0.003, 95%CI [− 0.02, 0.01, χ^2^c = 0.14, *p* > 0.05; model 4). The observed effects were unlikely to be driven by variation in day and night lengths as a bird migrates, as supplementary analysis found the same statistical relationships when using time proportional to shifting day and night lengths (supplementary table 1).

For every 1 day increase in migration departure date, the birds reduced their migration time by 7.68 h. Migration duration to the Patagonian shelf shortened as both mean moonlight illumination (β = − 15.63,0 95%CI [− 22.16, − 8.77], χ^2^c = 21.29, *p* < 0.001; Fig. 2; model 6) and autumn migration start date increased (β = − 0.32, 95%CI [− 0.39, − 0.25], χ^2^c = 74.89, *p* < 0.0001; Fig. 2; model 7), with the interactive effect being non-significant (β = − 0.23, 95% CI [− 0.82, 0.38], χ^2^c = 0.55, *p* > 0.5; model 7). This means that moonlit nights and late start date additively decreased migration duration, with the effect of moonlight not differing in birds migrating at different times.

Colony differences were included in all models and no significant differences were found in the number of hours spent flying per night (χ^2^c = 15.37, *p* > 0.5; model 2), the proportion of the night spent flying with the moon above the horizon (χ^2^c = 9.35, *p* > 0.5; model 3), night hours spent foraging (χ^2^c = 0.731, *p* > 0.5; model 4), stopover time (χ^2^c = 5.39, *p* > 0.5; model 5), foraging at stopovers (χ^2^c = 9.03, *p* > 0.5; model 6) or the mean chlorophyll ratio at stopovers compared to the over-wintering site (χ^2^c = 4.77, *p* > 0.5; model 10). Birds from Skomer were found to fly significantly less overall than birds from other colonies (β = − 0.27, 95% CI [− 0.46, − 0.08], χ^2^c = 54.85, *p* < 0.001; model 1), however this did not appear to affect their migration duration which was not significantly different to other colonies (β = 1.91, 95% CI [− 0.23, − 4], χ^2^c = 19.66, *p* > 0.05; Fig. 2; model 7). However, birds from the colony of Rum took a significantly longer migration duration (β = 4.76, 95% CI [2.07, 7.5], χ^2^c = 19.66, *p* < 0.01; Fig. 2; model 7), perhaps due to Rum being located furthest away from the over-wintering site.

For random effects, the intra-class correlation (ICC) for year differences was < 0.02, in all models indicating that only 2% of the total variation in stopover behaviour was attributable to year-level differences. Individual differences explained < 20% of total variance in all models, except for those investigating relationships with migration departure date and lay date (model 8), and with breeding period (model 9), where individual level difference explained 66% of the total variance.

## Discussion

In a seabird with substantial individual variation in lay dates^30^ we found that lay date predicted a sequence of knock-on phenological events, with later-laying birds departing on migration later, but taking less time to migrate and making fewer, shorter stopovers. This is consistent with the finding that late departing Cory’s shearwaters took a faster migration^22^ and experimental evidence that shearwaters with extended breeding periods still arrived promptly at the over-wintering grounds^12^. More widely, these results are consistent with the faster migrations observed to compensate for late departures across other long distance avian migrants^33–35^. We additionally found some flexibility in breeding behaviour, with later laying birds having shorter breeding periods (Fig. 1). This result suggests that earlier laying birds could provide a longer period of parental care to their offspring, which may be a factor for consideration in the well documented relationship between early lay dates and high breeding success in shearwaters and other avian taxa^36–38^. By shortening their breeding period to depart on migration, later laying birds most likely trade off the current reproductive attempt with both their own survival, and future reproductive attempts. This study presents novel evidence that individuals can minimise both breeding and migratory durations to regain time after laying later.

Perhaps to facilitate migratory catch-up, later-laying birds spent more time in flight during migration stints and flew more at night (Fig. 3). Whilst a previous analysis of migratory movements did not find increased activity in Manx shearwaters via moon illumination^32^, here we provide compelling evidence using a larger dataset for the exploitation of moonlight (Fig. 4). It appears that birds are using moonlight for migratory flight as opposed to foraging (Table 2), and migration duration shortening when birds experience higher levels of moonlight during migratory stints (Fig. 2). Birds could be using the moon for navigation purposes, using the moon as a heading indicator to maintain orientation over short periods^39,40^. It is more likely that birds are using the increased illumination to facilitate the use of visual cues related to flight control^25^. Manx shearwaters utilise shear-soaring or flap-gliding flight, both of which rely on the ability to assess accurately the distance to the sea surface^41,42^. Moonlight therefore allows for increased nights of flight, allowing later-laying birds to reach the over-wintering grounds in less time. Arriving at the wintering ground promptly appears sufficiently important that when they have little time, shearwaters will trade off a later departure for increased night flight. This flight likely occurs on low fuel levels, as later laying shearwaters take fewer stops for refuelling.

**Table 2:** A table representing all mixed-effects models present in this analysis (1–10).

Response	No	Coefficient	Effect Size	Confidence Intervals	χ^2^	*p* Value
Number of hours per day/night spent flying	1	Intercept	7.016	6.670, 7.344	–	–
		Night	− 3.827	− 4.142, − 3.496	2656.1	< 0.0001***
		Departure date (days)	0.001	− 0.005, 0.009	72.29	< 0.0001***
		Night * departure date	0.028	0.019, 0.036	42.525	< 0.0001***
		Colony (Lundy)	0.000	− 0.290, 0.287	54.846	> 0.05
		Colony (Ramsey)	0.218	− 0.281, 0.762	54.846	> 0.05
		Colony (Rum)	− 0.031	− 0.287, 0.234	54.846	> 0.05
		Colony (Skomer)	− 0.267	− 0.464, − 0.079	54.846	< 0.0001***
		Intercept	2.106	1.527, 2.676	–	–
		Moon	3.245	2.584, 3.900	1237.1	< 0.0001***
		Departure date (days)	0.015	0.001, 0.028	35.419	< 0.0001***
		Moon * departure date	0.018	0.001, 0.037	3.88	> 0.05
		Colony (Lundy)	0.384	− 0.101, 0.880	15.369	> 0.05
		Colony (Ramsey)	0.569	− 0.300, 1.422	15.369	> 0.05
		Colony (Rum)	0.256	− 0.167, 0.698	15.369	> 0.05
		Colony (Skomer)	− 0.191	− 0.536, 0.127	15.369	> 0.05
Proportion of Night Spent Flying	3	Intercept	0.293	0.271, 0.316	–	–
		Moon up	0.082	0.069, 0.095	140.52	< 0.0001***
		Colony (Lundy)	− 0.002	− 0.038, 0.033	9.348	> 0.05
		Colony (Ramsey)	0.021	− 0.042, 0.082	9.348	> 0.05
		Colony (Rum)	0.009	− 0.020, 0.042	9.348	> 0.05
		Colony (Skomer)	− 0.024	− 0.049, − 0.001	9.348	> 0.05
Number of hours per night spent foraging	4	Intercept	6.134	5.604, 6.672	–	–
		Moon	− 2.846	− 3.369, − 2.298	1069.3	< 0.0001***
		Departure date	0.000	− 0.011, 0.009	0.822	> 0.05
		Moon* departure date	− 0.003	− 0.017, 0.011	0.135	> 0.05
		Colony (Lundy)	0.280	− 0.214, 0.779	0.731	> 0.05
		Colony (Ramsey)	0.196	− 0.582, 1.014	0.731	> 0.05
		Colony (Rum)	− 0.133	− 0.603, 0.308	0.731	> 0.05
		Colony (Skomer)	− 0.023	− 0.358, 0.320	0.731	> 0.05
Stopover Time (days)	5	Intercept	15.158	12.387, 18.060	–	–
		Departure date	− 0.156	− 0.218, − 0.097	28.539	< 0.0001***
		Colony (Lundy)	0.362	− 2.124, 2.778	5.391	> 0.05
		Colony (Ramsey)	− 2.239	− 7.278, 2.929	5.391	> 0.05
		Colony (Rum)	2.164	− 0.058, 4.588	5.391	> 0.05
		Colony (Skomer)	0.892	− 0.821, 2.641	5.391	> 0.05
Foraging at stopovers (mean number of hours)	6	Intercept	12.556	11.462, 13.608	–	–
		Departure date	0.010	− 0.007, 0.030	0.300	> 0.05
		Colony (Lundy)	1.524	0.342, 2.781	9.034	> 0.05
		Colony (Ramsey)	1.271	− 0.930, 3.229	9.034	> 0.05
		Colony (Rum)	1.090	0.010, 2.198	9.034	> 0.05
		Colony (Skomer)	0.638	− 0.132, 1.495	9.034	> 0.05
Migration duration (days)	7	Intercept	50.217	45.680, 54.410	–	–
		Mean moon	15.633	− 22.155, − 8.768	21.285	< 0.0001***
		Departure date	− 0.323	− 0.390, − 0.249	74.892	< 0.0001***
		Mean moon * departure date	− 0.228	− 0.816, 0.378	0.546	> 0.05
		Colony (Lundy)	− 1.735	− 4.625, 1.180	19.659	> 0.05
		Colony (Ramsey)	− 0.656	− 6.857, 5.498	19.659	> 0.05
		Colony (Rum)	4.757	2.073, 7.501	19.659	< 0.01**
		Colony (Skomer)	1.905	− 0.231, 4.000	19.659	> 0.05
Departure Date (days)	8	Intercept	5.566	0.900, 10.207	–	–
		Lay date	0.566	0.372, 0.749	27.354	< 0.0001***
Breeding period (days)	9	Intercept	131.341	126.716, 135.954	–	–
		Lay date	− 0.441	− 0.645, − 0.248	18.898	< 0.0001***
Mean chlorophyll ratio (stopover/over-wintering)	10	Intercept	0.396	0.323, 0.475	–	–
		Departure date	− 0.005	− 0.007, − 0.003	42.316	< 0.0001***
		Colony (Lundy)	− 0.051	− 0.127, 0.029	4.774	> 0.05
		Colony (Ramsey)	− 0.051	− 0.228, 0.145	4.774	> 0.05
		Colony (Rum)	0.013	− 0.052, 0.081	4.774	> 0.05
		Colony (Skomer)	− 0.037	− 0.090, 0.014	4.774	> 0.05

For each coefficient, confidence intervals and effect sizes were obtained through bootstrapping, whilst chi-squared and p values were obtained through likelihood ratio tests. Significance levels are indicated by the number of asterisks (< 0.0001 ‘***’, < 0.001 ‘**’, < 0.01 ‘*’).

Phenological variation in migration start among individuals was coupled with behaviour at stopover sites, with earlier departing birds having longer stopovers. One explanation for this could be that late birds increase their fuel loading prior to migration to fly longer migratory stints with minimal stopovers, whilst early birds refuel along the way^14,43^. However, as foraging opportunities for later departing birds will be seasonally declining at the breeding site, this seems unlikely^44,45^. Additionally, both early and late departing birds foraged for a comparable number of hours whilst at stopover sites, suggesting late birds did not fuel load more prior to migration. We instead found that stopover sites for early departing birds had higher chlorophyll concentrations relative to the over-wintering grounds than the stopovers of late departing birds. Therefore, for later migrating birds, stopover sites in the northern hemisphere may be less profitable than the over-wintering grounds and they may therefore head across such sites more rapidly. Meanwhile, earlier departing birds, once released from chick provisioning duty, can exploit foraging opportunities along the way. With the maximum recorded stopover length reaching 22 days, we suggest that stopover sites for earlier migrating birds may not simply be for refuelling purposes but may also represent the best foraging locations available at that time. Birds that lay later may therefore miss the opportunity to exploit stopover sites that provide good foraging opportunities during the intermediate seasons. Whilst in this analysis we have explored how variation in seasonal productivity may determine migratory behaviour, other environmental variables may result in stopovers or deviations in migration, such as winds or storms, which should be considered in future studies to fully understand individual migratory strategies^46–48^.

To conclude, here we demonstrate that individuals can flexibly adjust their migratory behaviour to compensate for phenological constraints that arise from breeding. Despite leaving later on migration, late-laying birds are able to catch up via flying more at night and stopping over less to forage. Whilst our study shows the importance of how the timing of events can determine individual behaviour, it highlights the value of understanding the determinants of phenology in long-distant migrants.

## Methods

### Ethical statement

All animal handling work and biotelemetry procedures were ethically approved by the University of Oxford Animal Welfare and Ethical Review Board (AWERB) and permitted by landowners and relevant organisations. All bird handling and device deployments were conducted under British Trust for Ornithology (BTO) licensing and all procedures followed ethical regulations for burrow nesting seabirds. Additionally, animal work followed ARRIVE guidelines in designing and implementing a multi-year study recording non-experimental behaviour, assuring samples were randomised and outcome measures were determined prior to analysis.

### Fieldwork

From 2006 to 2020, 774 geolocator (GLS) archival devices were attached to Manx shearwaters caught at their nest during the breeding season to record their migratory and movement behaviour year-round. Devices were attached using cable ties to custom built plastic leg rings and removed for download in subsequent breeding seasons. Fieldwork was carried out on five UK breeding colonies on Rùm (57.01° N, 6.33° W), Skomer (51.74° N, 5.29° W), Ramsey (51.74° N, 5.29° W), Copeland (54.68° N, 5.53° W), and Lundy (51.18° N, 4.67° W). Handling time was kept to a minimum (5–15 min).

Multiple models of combined immersion and light loggers were used as part of this study: BAS Mk 6, 9, 15, 19, 4083 (2.5 g; 0.6% of average bird mass), BAS Mk 13, 14, 18 (1.5 g; 0.38% of average bird mass) and MigrateTech intigeo C330, (3.3 g; 0.83% of average bird mass), C250 (2.5 g; 0.6% of average bird mass), C65, C65-Super (1 g; 0.25% of average bird mass). On Skomer Island lay dates were obtained through daily nest checks, totalling 105 nest-years across all years. Following the removal of birds who did not lay an egg, or did not successfully fledge a chick, this left a total of 90 lay dates for analysis.

### Data processing

All data processing was carried out in R Studio Version 4.1.2^49^. Bi-daily positions were calculated using GLS-recorded light data through the R package geolight, where latitude is defined by day length, and longitude by the shifting timing of midday^50^. We used a sun elevation angle of − 4.5° to minimise the number of positions over land, using latitude against time plots to best calibrate breeding season latitudes with that of the colony^31^. Migration start and end dates were determined via visual inspection of longitudinal changes between August and December, as in supplementary 1. Longitude, smoothed using a 3-day rolling mean, was used to determine phenology since it is not subject to equinox error. As there are differences between devices in immersion sensitivity, we only used a subsample of consistent immersion data (from 2007 to 2016). Immersion was sampled every 6 s by the GLS device and summed every 10 min to create a summary score ranging from 0 (dry for the whole period) to 200 (wet for the whole period). To analyse patterns of activity, data were filtered to remove tracks with incomplete migrations. This left 351 complete migrations which has immersion data for analysis from 186 individuals.

Given the associated error with geolocator position (especially around the equinox when shearwaters migrate)^51,52^, to identify stopovers, we used the logger’s immersion data to determine which days were associated with fewer hours of flight. The number of dry ten-minute bins was summed per day to indicate the amount of time spent in flight. A mixture model was then implemented from the R package mixtools^53^ using an EM algorithm for normal distributions to identify two discrete distributions in the number of flight hours per day (see supplementary materials 2). All bird-days were included in one model as we expected stopovers to be characterised by a low number of flight hours regardless of the individual bird, and including more migratory days improves parameter estimation and provides better separation of stopovers. As there was slight overlap between distributions, stopovers were assigned to bird-days with a > 0.66 probability of fitting into the peak with fewer flight hours. Following the assignment of stopovers, the total number of stopover days was summed for each track. The number of hours spent foraging at stopovers and during migration was calculated per day. Foraging was classified as ten-minute bins with intermediate immersion scores (between and 1 and 199), a method that has been applied to study foraging in Manx shearwaters^12,28^ and previously been validated using co-deployed Time Depth Recorder (TDR) data during breeding^54^.

To investigate flight behaviour whilst birds are migrating, stopover periods were excluded, so as to assess periods where birds were actively travelling. Days and nights were first separated using the devices’ light data. Moon illumination was obtained from the R package lunar^55^and moon rise and set times were obtained from the R package suncalc^56^ using the light curve-calculated positions from the GLS data. However, the locations were not accurate enough for determining cloud cover or meteorological conditions for each night. To ensure that any moon effects were not caused by weather, we examined whether flight behaviour could be attributed to moon state and risen status, which cannot be confounded with meterological conditions. Given that moon rise and set times do not show substantial variation on small spatial scales, light derived positions, despite their subjectivity to error, provided an adequate estimate of position for which to obtain daily moon timings. Lastly, chlorophyll a concentration (mg.m^-3^) was obtained for each stopover day using 1-day resolution NASA Aqua MODIS remotely sensed data^57^. The mean chlorophyll was calculated for a 0.08° N × 0.08° E grid around the mean latitude and longitude of each stopover over the days it occured, plus for the over-wintering site at the Patagonian shelf (− 46° N to − 36° N, − 66° E to − 54° E) for the same days^56^. A ratio was then calculated for each stopover (mean stopover chlorophyll/over-wintering chlorophyll) to determine the relative productivity of stopover sites to the over-wintering site.

### Statistical analysis

To test the effects of autumn migration start date and moon factors on migration duration, stopover activity, the mean chlorophyll ratio and hours of flight/foraging, we used linear mixed-effects models fitted with maximum likelihood^58^. For the hours of flight/foraging, rather than code the responses as 0 s or 1 s for each immersion bin, we summed the bins per bird night which provided a near-continuous response variable. Hence, we proceeded with a gaussian general linear mixed model and checked the assumption of approximately normal residuals, which was met for all models. Colony was fitted as a fixed effect in all multi-colony models to account for potential differences in autumn migration metrics and ecological setting. We accounted for non-independence between years and individuals by including them as random intercepts in all models. To assess whether variation in time spent flying or foraging is independent of changes in day length during migration, we conducted a supplementary analysis. We fit the same models but used the proportion of hours in each day or night as the response variable. Significance was obtained using likelihood ratio tests between the full model and a null model with the parameter of interest dropped, implemented using the R package lme4^58^. Confidence intervals and effect sizes were generated using bootstrapping methods through the ‘arm’ package in R to resample model effects 1000 times^59^.

### Statement on inclusion

Our study involves scientists from a number of different countries and includes their diverse perspectives. Our field sites include scientists based at local sites. Research from these regions is additionally cited within this study. This work has also provided training opportunities for ECR scientists.

## Supplementary Information

Supplementary Information.

## Data Availability

Data is available on zenedo 10.5281/zenodo.11236852**.**
