# Supplementary material for: Night flight facilitates late breeding catch-up in a long-distance migratory seabird
Source: Sci Rep. 2024 Dec 30;14:31792. doi: 10.1038/s41598-024-82328-4 (PMC11685964; doi:10.1038/s41598-024-82328-4)
Supplement: Supplementary file 1 — Supplementary Information. [file 41598_2024_82328_MOESM1_ESM.docx]

## **Supplementary Materials:** Night flight facilitates late breeding catch-up in a long-distance migratory seabird

^1^ Department of Biology, University of Oxford, Mansfield Road, Oxford, OX1 3SZ, United Kingdom

^2^ Institut für Vogelforschung “Vogelwarte Helgoland”, An Der Vogelwarte 21, 26386, Wilhelmshaven, Germany

^3^ Department of Earth, Ocean and Ecological Sciences, University of Liverpool, Jane Herdman Building, L69 3GP, United Kingdom

^4^ Faroe Marine Research Institute Nóatún 1, P.O. Box 305, FO 110, Tórshavn, Faroe Islands

^5^ Norwegian Institute for Nature Research, Høgskoleringen, Trondheim, 7034, Norway

^6^ Zoological Society of London, London, NW1 4RY

^7^ ICON Science, Royal Melbourne Institute of Technology, La Trobe Street, Melbourne VIC 3000, Australia

^8^ British Geological Survey, MacLean Building, Benson Ln, Crowmarsh Gifford, Wallingford OX10 8ED, United Kingdom

^9^ Royal Society for the Protection of Birds, Pembrokeshire Coast National Park, Haverfordwest SA62 6PY

^10^ Department of Vertebrate Ecology and Zoology, Faculty of Biology, University of Gdańsk, Wita Stwosza 59, 80-308 Gdańsk, Poland

^11^ Graduate School of Environmental Studies, Nagoya University, Nagoya, 464-8601, Japan


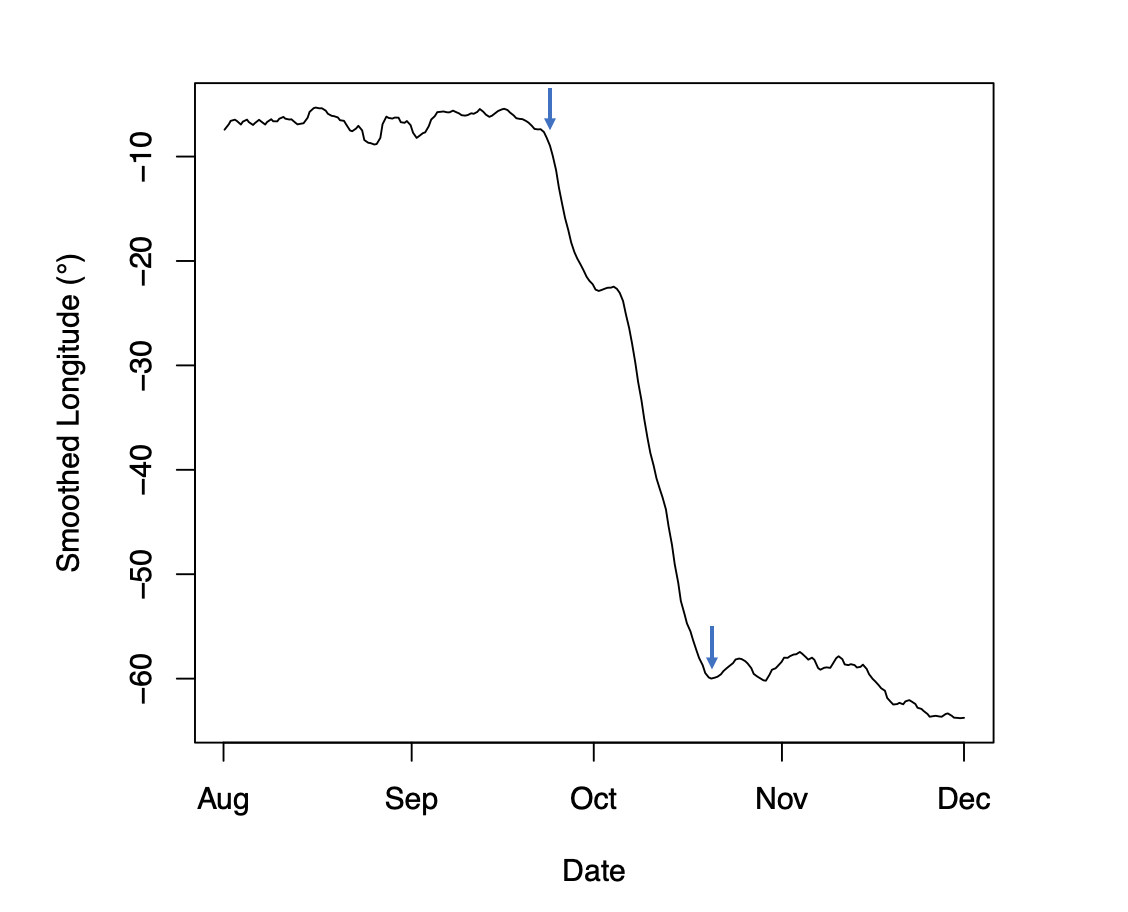


**Supplementary 1:** Determination of migration start and end dates using smoothed longitude. Longitude was smoothed using a rolling 3 day mean due to associated geolocator error. Arrows indicate visually assigned migration start and end dates. During the over-wintering period longitude continues to change as birds forage around the South American coast and down to the Patagonian shelf. This method identifies migration as the period in which longitude change is steepest, as this is the time when birds are actively migrating.


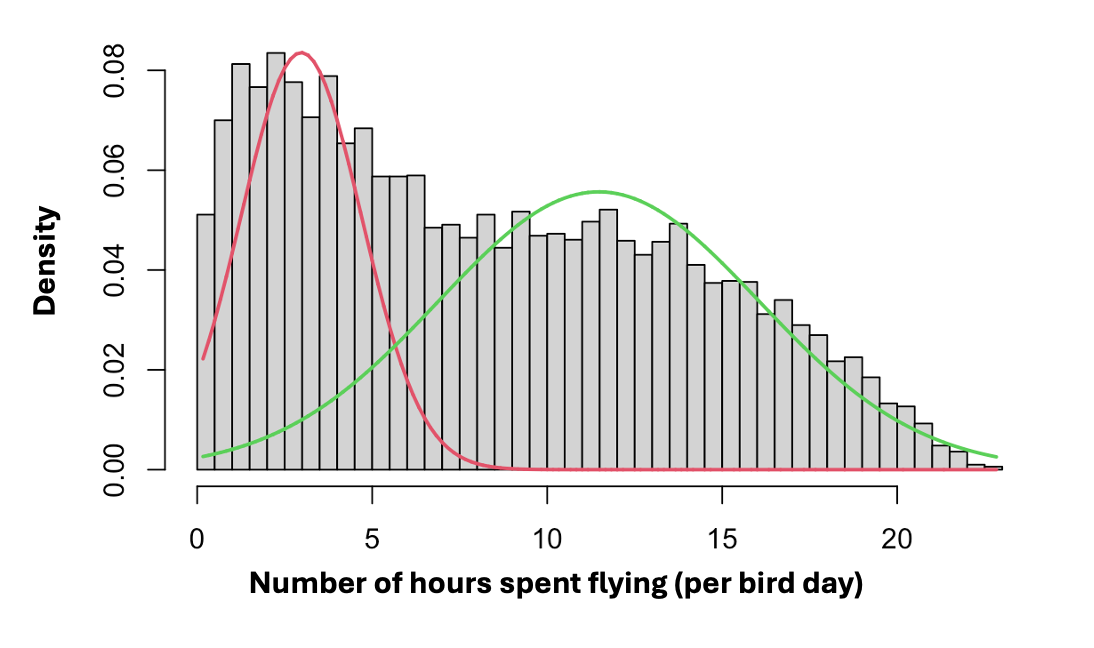


**Supplementary 2:** The determination of stopover days from geolocation immersion data during post breeding migration. An EM mixture model for univariate normal distributions was applied to the number of hours spent flying (per bird day) with data from all birds compiled. From the model, two distributions were distinguished: one corresponding to a peak with less recorded flight (red) and one with greater levels of immersion (green). Stopovers were assigned to bird days with a probability of >66% of belonging to the peak with lower flight time.

| **Response** | **No.** | **Coefficient** | **Effect Size** | **Confidence Intervals** | **χ^2^** | **p value** |
| --- | --- | --- | --- | --- | --- | --- |
| Proportion of Day/Night Spent Flying | 1 | *Intercept*  **Night**  **Departure date**    **Night* departure date**  Colony (Lundy)  Colony  (Ramsey)  Colony  (Rum)  **Colony**  **(Skomer)** | 0.538  -0.153  0.008  0.051  0.001  0.023  -0.002  -0.022 | 0.519, 0.557  -0.162, -0.145  -0.006, 0.023  0.034, 0.069  -0.025, 0.025  -0.018, 0.067  -0.026, 0.021  -0.039, -0.005 | -  34.605  68.142  1221.1  47.063  47.063  47.063  47.063 | -  < 0.0001***  < 0.0001***  < 0.0001***  >0.05  >0.05  >0.05  < 0.0001*** |
| Proportion of Night Spent Flying | 2 | *Intercept*  **Moon**  **Departure date**  Moon * departure date  Colony (Lundy)  Colony  (Ramsey)  Colony  (Rum)  Colony  (Skomer) | 0.243  0.359  0.027  0.038  0.034  0.049  0.021  -0.018 | 0.214, 0.273  0.341, 0.379  0.001, 0.052  -0.002, 0.075  -0.010, 0.079  -0.020, 0.119  -0.018, 0.063  -0.047, 0.013 | -  1320.2  29.882  3.94  15.475  15.475  15.475  15.475 | -  < 0.0001***  < 0.0001***  >0.05  >0.05  >0.05  >0.05  >0.05 |
| Proportion of Night Spent Foraging | 3 | *Intercept*  **Moon**  Departure date  Moon * departure date  Colony (Lundy)  Colony  (Ramsey)  Colony  (Rum)  Colony  (Skomer) | 0.579  -0.274  -0.011  -0.005  0.034  0.010  -0.006  0.002 | 0.543, 0.613  -0.292, -0.258  -0.034, 0.015  -0.039, 0.028  -0.016, 0.083  -0.063, 0.087  -0.049, 0.039  -0.028, 0.034 | -  987.12  2.287  0.101  2.641  2.641  2.641  2.641 | -  < 0.0001***  >0.05  >0.05  >0.05  >0.05  >0.05  >0.05 |
| Foraging at stopovers (proportion) | 4 | *Intercept*  Departure date  Colony (Lundy)  Colony  (Ramsey)  Colony  (Rum)  Colony  (Skomer) | 0.540  0.012  0.064  0.057  0.046  0.027 | 0.508, 0.574  -0.008, 0.032  0.015, 0.110  -0.029, 0.142  -0.001, 0.093  -0.008, 0.060 | -  1.368  8.829  8.829  8.829  8.829 | -  >0.05  >0.05  >0.05  >0.05  >0.05 |

**Supplementary table 1.** A table representing all supplementary mixed-effects models present in this analysis (1-4). Response variables are taken as a proportion of the day/night to account for variation in day and night length during migration. Migration start date was standardised (by centering and dividing each value by 2 standard deviations), so variables were on comparable scales. For each coefficient, confidence intervals and effect sizes were obtained through bootstrapping, whilst chi-squared and p values were obtained through likelihood ratio tests. Significant predictors are highlighted in bold and significance levels are indicated by the number of asterisks (< 0.0001 ‘***’, <0.001 ‘**’, <0.01 ‘*’)

|  | **Copeland**  **(n=78)** | **Lundy**  **(n=41)** | **Skomer**  **(n=174)** | **Ramsey**  **(n=6)** | **Rum**  **(n=52)** |
| --- | --- | --- | --- | --- | --- |
| **Migration Start Date** | 17^th^ September | 7^th^ September | 13^th^ September | 11^th^ September | 18^th^ September |
| **Migration End date** | 16^th^ October | 8^th^ October | 16^th^ October | 11^th^ October | 21^st^ October |
| **Migration Duration** | 29.34±6.53 | 30.98 ±8.38 | 32.53±7.46 | 29.33±5.09 | 33.20±12.87 |
| **Stopover number** | 3.50±1.79 | 4.10±1.84 | 4.31±1.99 | 3.83±0.75 | 3.92±2.50 |
| **Total Stopover days** | 8.77±5.09 | 10.73±6.72 | 10.31±5.87 | 7.50±2.74 | 10.80±8.58 |
| **Stopover length** | 2.74 ± 1.73 | 2.78 ± 1.72 | 2.61 ±1.44 | 1.95± 0.58 | 2.87±1.46 |
| **Time between stopovers** | 6.28 ± 3.10 | 4.76 ±1.81 | 5.20 ±2.60 | 5.27±1.25 | 6.87±4.49 |
| **Continuous flight** | 0.46 ± 0.10 | 0.44 ± 0.07 | 0.44 ± 0.07 | 0.46± 0.07 | 0.46±0.07 |

**Supplementary table 2.** Summary statistics of autumn migration parameters. For all parameters we present the mean (of individual means) ± the standard deviation for each colony derived from 151 bird migrations.
